# The Association Between Smoking and Health-Related Quality of Life Among Chinese Individuals Aged 40 Years and Older: A Cross-Sectional Study

**DOI:** 10.3389/fpubh.2022.779789

**Published:** 2022-02-24

**Authors:** Xi Cheng, Chenggang Jin

**Affiliations:** School of Social Development and Public Policy, Beijing Normal University, Beijing, China

**Keywords:** smoking, health-related quality of life, adult smoking, quality of life, association

## Abstract

**Objective:**

The purpose of this study was to investigate the association between smoking and health-related quality of life among Chinese individuals aged 40 years and older.

**Method:**

Using a stratified multistage sampling method, data from 1,543 adults aged 40 years and older were obtained from a household survey conducted in eight provinces in China. The health-related quality of life was quantified based on the utility index obtained using a standardized instrument entitled “The European Five-Dimensional Health Scale (EQ-5D-5L).” Descriptive statistics were used to summarize the demographic characteristics and social factors of the sample according to smoking status. An instrumental variable (IV) probit model was used to estimate the association between smoking status and health-related quality of life.

**Results:**

Of the 1,543 participants, 485 (31.43%) were smokers and 1,058 (68.57%) were non-smokers. Smoking was negatively associated with the probability of having a higher quality of life (*p* < 0.01). For smokers, the average probability of having a higher quality of life was 11.65% lower than when they did not smoke.

**Conclusions:**

These findings suggest that smoking reduces health-related quality of life among Chinese individuals aged 40 years and older. Anti-smoking programs should consider this factor.

## Introduction

The tobacco epidemic is the greatest but preventable risk factor for human health. Approximately 8 million people worldwide die from smoking each year, and more than 80% of the 1.3 billion tobacco users worldwide live in low- and middle-income countries (1). According to the 2018 Global Adult Tobacco Survey (GATS), there are more than 300 million smokers in China, with 52.9% of Chinese male adults smoking (2). China's health, society, and economy are suffering due to tobacco consumption. More than 1 million people die from tobacco-related deaths in China each year. This number will continue to grow—to ~3 million by 2,050 if China does not act effectively to control its smoking epidemic (3).

In addition to health threats, smoking can also directly affect health-related quality of life (HRQoL) (4–7). As a comprehensive health evaluation index, quality of life is a self-assessment, that measures people's self-report of their physical state, mental function, social ability, and personal overall condition based on certain socioeconomic and cultural backgrounds and values.

It seems to be a common belief that smoking can help relieve stress and promote relaxation, thus creating the illusion that smokers have a much higher quality of life than non-smokers. However, accumulating evidence suggests that HRQoL is better among non-smokers and former smokers than among current smokers (8–10). The negative association between smoking and HRQoL has been demonstrated in several cross-sectional studies (11–13). Their results were further confirmed by longitudinal studies focusing on the association between smoking status and changes in HRQoL (14–17).

The association between smoking and HRQoL may have different manifestations in different countries, where the cultural context may be at play. Most related studies have been conducted in western countries, including European countries (18–21), the United States (22), and a few other countries (23–26). Although China is the largest tobacco producer and consumer in the world, few studies have systematically examined the association between smoking and HRQoL among Chinese individuals. Besides, smoking is a continuous behavior, and its process of causing harm to human health is long-term and chronic (27). As a result, smoking-related side effects may be more easily perceived in middle-aged and older adults than in younger adults. Therefore, while the current mainstream literature shows that smoking is negatively related to quality of life, it is still necessary to evaluate the impact of smoking on the quality of life of the Chinese population aged 40 years and above.

The challenges in studying the association between smoking status and HRQoL are sample self-selection and sampling bias. However, the possible endogeneity of the relationship between smoking status and quality of life has rarely been considered. Whether to smoke is a self-selective behavior that can be influenced by many factors. The omitted variables that may affect both smoking and HRQoL will make the results less credible.

Therefore, the objective of this study was to explore the association between smoking and HRQoL among Chinese individuals aged 40 years and older using an instrumental variable (IV) probit model.

## Materials and Methods

### Participants

Participants (*N* = 1,543) were Chinese individuals aged 40 years and older recruited in a household survey conducted in China between November 1, 2019, and October 30, 2020. A stratified multistage sampling method was used to select participants from 24 primary health care facilities. These 24 primary health care facilities were selected as follows: firstly, 8 provinces were selected in the east, middle, and west of China: Hebei, Heilongjiang, Shandong, Henan, Hubei, Sichuan, Guizhou, and Shaanxi. Then 2–4 primary health care institutions, including township health centers and community health service centers, were randomly selected in each province. From the areas of 24 primary health care facilities, ~100 households were randomly selected. To be eligible, participants from the 100 households had to live in local communities for at least 6 months, have a minimum age of 40 years, and be willing to participate in this study.

### Data Collection

Based on informed consent, the data were collected using anonymous paper and pencil tests. Participants completed questionnaires entitled “Questionnaire on the health of people over 40 years old and its influencing factors.” The validated interviewer-administered questionnaire mainly included (1) general household information, including household type, total household income, and expenditure; (2) basic personal information of household members, including gender, age, and education level; (3) smoking, smoking-related knowledge and chronic diseases of household members; and (4) self-care ability and quality of life of household members.

In this study, the investigators were designated by each investigation unit, and then the investigators were uniformly trained by the research team. The means of household inquiries were adopted by the investigators. Besides, each survey unit identified a contact person who was responsible for survey organization, implementation, quality control, and unified reporting to the subject group. Finally, the research team organized and coded the questionnaires in a unified manner. This study was approved by the ethics committee of the Capital Institute of Pediatrics, Beijing (ID: SHERLL2020017). And the study was conducted following the ethical principles of the Declaration of Helsinki.

### Measures

#### Dependent Variables

The dependent variable was “quality of life utility index.” The HRQoL was quantified based on the utility index obtained using a standardized instrument entitled “The European Five-Dimensional Health Scale (EQ-5D-5L).” The EQ-5D is easy to operate and easy to understand by the survey subject and has good reliability and validity. Therefore, it has been widely used in various research fields in many countries (28) and has become one of the widely used tools for measuring HRQoL. The EQ-5D survey includes five dimensions: mobility, self-care, usual activities, pain or discomfort, and anxiety or depression. An approved Chinese version of the EQ-5D-5L was used, and each level contained five possible responses indicating “no problems,” “slight problems,” “moderate problems,” “severe problems” and “unable to/extreme problems.” If “no problems” were reported for a given level, it was marked as level 1, whereas “unable to/extreme problems” was marked as level 5. The eq5d command in STATA (StataCorp, College Station, Texas, USA) computes an index value from individual responses to the EQ-5D-5L quality-of-life instrument. The EQ-5D index has an upper bound equal to 1 that indicates full health (as evidenced by “no problem” in all domains), whereas 0 represents death (29). To facilitate analysis, we dichotomized the EQ-5D index according to its mean value.

#### Independent Variable

The measure of smoking status came from responses to the following question: “Do you smoke now?” In the current study, the concept of smoking was defined as “having smoked at least 1 day in the past 30 days.” Response options were (1) No, (2) Yes, and (3) Have quit smoking. To facilitate statistical analysis, smoking status was dummy coded: (1) smoking: participants who smoke now; (2) non-smoking: participants who had never smoked or had quit smoking.

#### Covariates

Based on prior knowledge (30), covariates included age, gender, educational level, marital status, logarithm of household income, occupation, family size, health status, and province.

#### Instrumental Variable

Whether the increase in cigarette prices reduced the number of cigarettes smoked (PRS) was used as an instrumental variable.

The variables used in the regression analysis are listed and defined in Table 1.

**Table 1:** The definition and abbreviation of the variables in the model.

**Variable**	**Abbreviation**	**Definition**
Smoking status	Smoke	Binary variable scored 1 if participants smoke now and 0 otherwise
Quality of life utility index	Index	Binary variable scored 1 if the quality of life utility index is larger than its mean value and 0 otherwise
Age	Age	Continuous variable measured in years
Gender	Gender	Binary variable scored 1 for males and 0 for females
Educational level	Education	Categorical variable scored 1 for Illiterate, 2 for Primary School, 3 for Middle School, 4 for High School and Junior College, and 5 for College and above
Marital status	Marriage	Categorical variable scored 1 for Single, 2 for Married, and 3 for Divorced or Widowed
Logarithm of family income	Lnincome	Continuous variable
Occupation	Occupation	Categorical variable scored 1 for Party and government agencies or institutions staff/State-owned enterprises or private enterprise staff, 2 for Self-employed/freelance workers, 3 for Rural migrant workers/Rural local non-agricultural workers/Agriculture, forestry, animal husbandry and fishery workers, 4 for Retired workers, and 5 for Unemployed people
Family size	Family size	Categorical variable scored 1 for one, 2 for two, 3 for three, and 4 for four and above
Health status	Health status	Categorical variable scored 1 for 0–2 chronic diseases and 2 for 3 or more chronic diseases
Province	Province	Categorical variable scored 1 for Henan, 2 for Heilongjiang, 3 for Shandong, 4 for Hebei, 5 for Sichuan, 6 for Hubei, 7 for Guizhou, and 8 for Shaanxi
Whether the increase in cigarette prices reduced the number of cigarettes smoked	PRS	Binary variable scored 1 if the increase in cigarette prices reduced the number of cigarettes smoked and 0 otherwise

### Data Analysis

#### Descriptive Analysis

Descriptive statistics were used to summarize the demographic characteristics and social factors of the sample by smoking status. Characteristics of smokers were compared with those of non-smokers using chi-square tests for categorical variables and Kruskal–Wallis one-way ANOVA tests for continuous variables. Descriptive data are presented as the mean for continuous variables and as percentages for categorical variables.

#### Effect Estimation

To investigate the association between smoking status and HRQoL, an instrumental variable (IV) probit model was used to control for potential endogeneity problems. Whether the increase in cigarette prices had an effect on the number of cigarettes smoked (PRS) was used as an instrumental variable. It was chosen because it is expected to be correlated with smoking behavior but not directly affect quality of life, thus satisfying the instrumental variable exogeneity requirement. Covariates included age, gender, educational level, marital status, logarithm of annual household income, occupation, family size, health status, and province. The province variable was entered into the model as a dummy variable to give the province a specific intercept to capture sample clustering.

The parameter estimates from the IV probit were further used by marginal analysis to estimate the average treatment effect on the treated (ATET) of smoking on HRQoL. ATET is the estimated average difference of the treatment and control potential outcomes in the treated population. ATET is useful when there is interest in the quantification of the treatment effect in observational studies in which no definite parameter can be used. Therefore, ATET was calculated to obtain more intuitive and practical results.

The instrumental variable (IV) probit model is constructed below as:


(1) $$\begin{array}{l} Index_{i}\;=\;\{\begin{array}{c} \;1\;if,\;Index_{i}^{*}\geq 0.95\;\\ 0\;if,\;otherwise\; \end{array} \end{array}$$

(2) $$\begin{array}{lll} Index_{i}^{*}\; & = & \;\beta_{1}Smoke_{i}\;+\;\gamma Z\;+\;\mu_{i} \end{array}$$

(3) $$\begin{array}{lll} Smoke_{i}\; & = & \;\pi_{1}Z\;+\;\pi_{2}I\;+\;\alpha_{i} \end{array}$$

Here, *Index*_*i*_ refers to the quality of life utility index of the respondents and $Index_{i}^{*}$ represents the latent variable of the quality of life utility index in Equation (1). *Smoke*_*i*_, the independent variable of interest, is binary. β_1_ is the coefficient of interest, which provides the estimated effect of smoking on HRQoL. *Z* is a vector of demographic and socioeconomic variables. *I* represents the instrumental variable. γ, π_1_ and π_2_ are the vectors of parameters for the control variables that need to be estimated. μ_*i*_ and α_*i*_ are normally distributed error terms in the equation and *i* denotes an individual respondent.

All statistical analyses were performed using Stata (version 16.0; StataCorp, College Station, Texas, USA). Values of *p* < 0.05 were considered statistically significant.

## Results

### Sample Characteristics

The characteristics of subjects are summarized in Table 2. Of the 1,543 participants, 485 (31.43%) were smokers, and 1,058 (68.57%) were non-smokers. The average age of smokers was 55.51, whereas that of non-smokers was 53.86 (*p* = 0.005). The proportion of smokers among males was 54.41%, whereas the proportion among females was 7.76% (*p* < 0.001). The mean value of the logarithm of family income for smokers was 11.03 compared to 11.14 for non-smokers (*p* = 0.006). There was no significant difference concerning educational status (*p* = 0.268), marital status (*p* = 0.358), occupations (*p* = 0.098), and family size (*p* = 0.394) between smokers and non-smokers, whereas there were significant differences in the distribution of provinces (*p* < 0.001). Among the participants with two or fewer chronic diseases, 30.91% were smokers and 69.09% were non-smokers (*p* = 0.009).

**Table 2:** Sample characteristics according to smoking status (*N* =1,543).

**Characteristics**	**Smokers**	**Non-smokers**		
	***N* (%)/Mean**	***N* (%)/Mean**	**χ2/F**	***P*-value**
Age	55.51	53.86	7.880	0.005
Gender			389.301	<0.001
Male	426 (54.41)	357 (45.59)		
Female	59 (7.76)	701 (92.24)		
Marital status			2.054	0.358
Single	6 (35.29)	11 (64.71)		
Married	419 (30.83)	940 (69.17)		
Divorced/widowed	60 (36.14)	106 (63.86)		
Education			5.195	0.268
Illiterate	18 (23.38)	59 (76.62)		
Primary school	64 (36.78)	110 (63.22)		
Middle school	124 (31.47)	270 (68.53)		
High school/Junior college	141 (32.27)	296 (67.73)		
University/College and above	135 (30.00)	315 (70.00)		
Province			26.484	<0.001
Henan	25 (22.52)	86 (77.48)		
Heilongjiang	84 (25.23)	249 (74.77)		
Shandong	21 (23.86)	67 (76.14)		
Hebei	63 (36.42)	110 (63.58)		
Sichuan	47 (31.76)	101 (68.24)		
Hubei	51 (29.48)	122 (70.52)		
Guizhou	166 (39.15)	258 (60.85)		
Shaanxi	28 (30.11)	65 (69.89)		
Lnincome	11.03	11.14	7.530	0.006
Occupation			7.829	0.098
1	173 (31.17)	382 (68.83)		
2	120 (34.88)	224 (65.12)		
3	27 (40.91)	39 (59.09)		
4	68 (26.25)	191 (73.75)		
5	94 (31.44)	205 (68.56)		
Family Size			2.987	0.394
1	35 (39.33)	54 (60.67)		
2	167 (31.51)	363 (68.49)		
3	137 (31.42)	299 (68.58)		
4	144 (30.06)	335 (69.94)		
Health status			6.907	0.009
1	464 (30.91)	1,037 (69.09)		
2	21 (50.00)	21 (50.00)		
Total	485 (31.43)	1,058 (68.57)		

### The Association Between Smoking and HRQoL

The middle column in Table 3 shows the average marginal effects of the probit regression model. The sign of the smoke variable was negative and statistically significant. Smoking decreases the probability of having a higher quality of life by 7.50 percent.

**Table 3:** Effect of smoking on HRQoL: IV probit model.

	**Dependent variables: quality of life utility index**	
	**Probit**	**IV-Probit**
**Smoking status (reference group: Non-smoking)**		
Smoking	−0.075^**^ (0.027)	−0.496^**^ (0.186)
Age	−0.006^***^ (0.001)	−0.024^***^ (0.005)
**Gender (reference group: female)**		
Male	0.061^*^ (0.024)	0.344^**^ (0.122)
**Education level (reference group: illiterate)**		
Primary school	0.004 (0.052)	0.023 (0.197)
Middle school	0.054 (0.051)	0.216 (0.198)
High school/Junior college	0.015 (0.054)	0.054 (0.208)
University/College and above	0.026 (0.059)	0.093 (0.223)
**Marital status (reference group: single)**		
Married	0.035 (0.097)	0.155 (0.360)
Divorced/widowed	0.019 (0.097)	0.112 (0.371)
Lnincome	0.004 (0.015)	0.012 (0.062)
**Occupation (reference group: 1)**		
2	0.014 (0.030)	0.074 (0.117)
3	0.050 (0.048)	0.241 (0.195)
4	−0.033 (0.035)	−0.131 (0.131)
5	−0.037 (0.036)	−0.132 (0.131)
**Family size (reference group: 1)**		
2	0.071 (0.053)	0.273 (0.183)
3	0.040 (0.054)	0.148 (0.186)
4	0.033 (0.056)	0.114 (0.191)
**Health status (reference group: 1)**		
2	−0.210^**^ (0.077)	−0.660^**^ (0.218)
_cons	1.700^*^ (0.787)	1.699^*^ (0.809)
*N*	1,502	1,502

*Province omitted from the table. Standard errors in parentheses*.

* *p < 0.05*,

** *p < 0.01*,

*** *p < 0.001*.

The right column in Table 3 presents the estimated results of the IV probit regression model. As expected, the results show that smoking was negatively correlated with the probability of having a higher quality of life (*p* < 0.01). As shown in Table 4, the estimated ATET of −0.1165 implies that for those who smoked, the average probability of having a higher quality of life would be 11.65 percent lower than it would be if they had not smoked. This result is higher than the 7.50 percent obtained by the probit regression model.

**Table 4:** ATET estimates of smoking on the HRQoL: IV probit model.

	**Margin**	**Unconditional Std. Err**.	**z**	**P>z**	**(95% Conf. Interval)**	
**ATET**						
Smoke (1 vs. 0)	−0.1165	0.038	−3.06	0.002	−0.191	−0.042

In Table 3, from the estimated results of the explanatory variables in the IV probit model, the effects of age and gender on HRQoL were significant at the 0.1 and 1% levels, respectively. The coefficient for age indicates that increases in age lower the probability of having a higher quality of life. If the subject was male, the probability of his having a higher quality of life was greater. Among the province variables, the effects of Shandong, Hebei, and Shaanxi on HRQoL were significant at the 1, 5, and 5% levels, respectively.

## Discussion

Our study indicated that smoking led to a lower probability of having a higher quality of life. For smokers, the average probability of having a higher quality of life was 11.65 percent lower than when they did not smoke.

These compelling findings confirm previous findings reported from other countries such as the UK (31, 32), USA (22), Spain (19, 33), Canada (4) and Turkey (8). The consistent findings when using different tools to measure HRQoL reinforces the conclusion that smoking is negatively associated with HRQoL (11, 34, 35). According to Toghianifar et al. (36), smokers scored lower than non-smokers in terms of general health, social functioning, role-emotional and mental health, whereas recent quitters had significantly improved role-emotional and mental health than those who had continued smoking or those who became smokers.

The reasons for the observed negative association between smoking and HRQoL can be attributed to the following aspects. First, smoking increases the risk of non-communicable diseases, including cancers and cardiovascular and respiratory diseases (37). Adults with more health diseases have worse quality of life (26). Second, smoking was found to be associated with increased odds of depression (38) and more clinically significant fatigue (39). Third, the substances inhaled in cigarettes are related to muscle weakness and decreased vitality (40). Forth, the EQ-5D used in the present study is a comprehensive measurement of HRQoL in terms of mobility, self-care, usual activities, pain or discomfort, and anxiety or depression. In the long term, smoking would affect the five dimensions of the EQ-5D, thereby reducing HRQoL (14).

Besides smoking status that was previously discussed, age and gender were found to be independent variables of a lower HRQoL. This finding suggests that smoking intervention programs might be targeted for specific populations, such as men in particular age groups who are current smokers, in order to better improve HRQoL.

Popular belief has it that quitting will decrease HRQoL—because individuals believe it interferes with relationships or produces a loss of smoking related pleasure (such as reducing stress or promoting relaxation). However, the current study indicated that smoking did not improve HRQoL as one would expect. This result contributes to the knowledge of the association between smoking and HRQoL. Knowledge of this association is useful for two reasons: (1) to assist the economic evaluation of cessation programs by providing a more direct measure of health outcomes than the cessation itself; (2) to provide a good reason for individuals to quit smoking.

The strengths of this study are as follows. First, it was based on a large and nationally representative sample of middle-aged and older Chinese individuals. We were able to examine the association between smoking and HRQoL and control many potential confounding factors. The large sample size enabled sufficient power for statistical inference. Second, we used the IV probit regression model to address selection bias. The estimated results of the IV probit regression model were higher than the estimated results of the probit regression model, indicating that the probit model might underestimate the effect of smoking on HRQoL because of selection bias.

There are several limitations to the present study. First, we relied on self-report measures, which may be subject to recall bias and social desirability effects. Second, the study was cross-sectional in design, thus making it hard to obtain any conclusions regarding exact cause-and-effect relationships. Longitudinal data may be needed to further explore the causal relationship between smoking and HRQoL. Third, the generalizability of our results to other populations is limited because we focused on China, and other countries may be different due to ethnic differences.

## Conclusion

Findings from the current study suggest that for smokers, the average probability of having a higher quality of life was 11.65% lower than when they did not smoke. Emphasizing that smoking will lead to a lower quality of life may help guide smokers to consciously quit smoking. Therefore, it is necessary for anti-smoking campaigns to clearly point out the negative effect of tobacco use on HRQoL.

## Data Availability Statement

The datasets presented in this article are not readily available because data involves personal privacy issues. Requests to access the datasets should be directed to Xi Cheng, 15107100093@163.com.

## Author Contributions

XC: conceptualization, data curation, writing - original draft, and writing - review & editing. CJ: conceptualization, funding acquisition, investigation, supervision, and writing - review & editing. All authors contributed to the article and approved the submitted version.

## Conflict of Interest

The authors declare that the research was conducted in the absence of any commercial or financial relationships that could be construed as a potential conflict of interest.

## Publisher's Note

All claims expressed in this article are solely those of the authors and do not necessarily represent those of their affiliated organizations, or those of the publisher, the editors and the reviewers. Any product that may be evaluated in this article, or claim that may be made by its manufacturer, is not guaranteed or endorsed by the publisher.
